# Intersectional Invisibility in Women’s Diversity Interventions

**DOI:** 10.3389/fpsyg.2022.791572

**Published:** 2022-05-25

**Authors:** Chuk Yan E. Wong, Teri A. Kirby, Floor Rink, Michelle K. Ryan

**Affiliations:** ^1^Faculty of Economics and Business, University of Groningen, Groningen, Netherlands; ^2^Department of Psychology, University of Exeter, Exeter, United Kingdom; ^3^Department of Psychological Sciences, Purdue University, West Lafayette, IN, United States; ^4^Global Institute for Women’s Leadership, The Australian National University, Canberra, ACT, Australia

**Keywords:** intersectionality, multiple identities, diversity intervention, inclusion, gender, race

## Abstract

Many diversity interventions for women are ineffective. One reason for this may be that the field that diversity interventions are usually based on, the social sciences, often do not consider intra-group differences among women. Specifically, differences by racialization may be excluded from such diversity interventions. The present research examines whether racially marginalized women have different diversity interventions needs than White women, and whether organizations are less likely to represent those needs (i.e., intersectional invisibility). Across an open-ended coding (*n* = 293) and a ranking study (*n* = 489), Black women noted a need to incorporate intersectional differences, Asian women prioritized methods to address challenges to their authority, and White women indicated a need to address agency perceptions. Improving work-life balance and networks was a shared concern among participants, though we theorized different racially gendered reasons for why these intervention needs are relevant to each group. In Study 3 (*n* = 92 organizations), we analyzed organizations’ websites using word count and textual analysis. Organizations— including the Education, Science, and Research sector— most readily advocated for women through enhancing agency. They were also less likely to mention dealing with perceptions of excessive agency or addressing intersectional considerations. The organizations broadly mentioned other marginalized groups besides women, but rarely did they do so intersectionality. Taken together, our findings demonstrate different intervention priorities across differently racialized groups. We found evidence of intersectional invisibility where organizations were more likely to address agency-enhancing intervention needs while failing to include other intervention needs relevant for Black women and Asian women. We discuss the implications of these findings for organizations, in general, as well as potential implications for the field of academic social sciences.

## Introduction

More women are entering the labor market than ever before (Eurostat, 2020). Yet, gender disparities in career advancement remain. Compared to men, women are still underrepresented in the labor market, paid less, and relegated to traditionally lower-paid work sectors (European Commission, 2021). In academic social sciences, particularly, women are paid less and are highly underrepresented in tenured positions or positions of power, despite increased representation in junior academic positions (e.g., Catalyst, 2020). These inequalities widen when considering racially marginalized women, who show lower rates of labor market participation, higher rates of unemployment, and more frequent experiences of discrimination than White women (ENAR, 2017; Gezici and Ozay, 2020).

In response to these inequities, organizations frequently implement diversity interventions (e.g., Shortland, 2011; Annabi and Lebovitz, 2018; Pietri et al., 2019). These diversity interventions aim to enhance participants’ professional development and prominence, as well as make working conditions more inclusive and equitable (Leslie, 2020). Women are often the target of these diversity interventions, where the goal is to help women overcome the gendered barriers that they face. However, perceptions of gender and race are intertwined, where gender is often interpreted together with one’s race and vice versa (Crenshaw, 1991; Chavez and Wingfield, 2018; Mukkamala and Suyemoto, 2018). Despite the co-constitution of gender and race, the differences in how racialization affects racially marginalized women and how it results in different needs for successful interventions, may not be incorporated in the content for these diversity interventions to fully support these women.

Indeed, even within the field of social sciences from which these diversity interventions are frequently based on, there are vulnerabilities that racially marginalized women uniquely experience that often remain at the margins. While there have been gains on the basis of gender in academia, less progress seems to have been made on the basis of race (Bhopal, 2018, 2020; Gause, 2021). Foreign women in academia strongly describe being hidden from view in academic studies and from the professional work floor (e.g., Strauβ and Boncori, 2020; Muradoglu et al., 2021). Even when these experiences come to light, they are often unaddressed due to the strong endorsement of meritocracy and colorblindness in academic institutions (Gvozdanović and Bailey, 2020; Azhar and McCutcheon, 2021). Therefore, overlooking the overlap between racialization and gender within the social sciences is presumably transferred onto the product of diversity intervention themselves.

Racially marginalized women may thus experience ‘intersectional invisibility’ in these diversity interventions for women, where a person with multiple subordinate group identities are rendered “invisible” relative to those with a single subordinate identity (Purdie-Vaughns and Eibach, 2008). In this research, we examine how a form of intersectional invisibility may be present in diversity interventions for women. We do this by first exploring whether there are racialized differences in what women consider to be beneficial for them in a diversity intervention, that is, if there are differences in their intervention needs. Second, we examine how these intervention needs are respectively represented among organizations.

### Considering Multiple Marginalizations in Diversity Interventions for Women

The dominant approach to researching diversity views oppression unidimensionally, focusing on single dimensions of oppression at a time (e.g., racism, sexism; Gopaldas and DeRoy, 2015; Breslin et al., 2017; Moradi, 2017). When designing diversity interventions for women and monitoring their impact, this unidimensional focus on gender overlooks and perpetuates two problems: (1) racially marginalized women may be excluded from diversity interventions for women, because women are implicitly racialized as White (Ghavami and Peplau, 2013; Thomas et al., 2014), and (2) even when racially marginalized groups are considered, they are often seen as men or, if any intersectional praxis is taken, Black women are studied and other racially marginalized women are rendered invisible (Fernandez, 2007; Deliovsky and Kitossa, 2013).

### The Prototypicality of White Women and Atypicality of Racially Marginalized Women

Diversity interventions for gender are intended to tackle gendered barriers and stereotypes. For women, these stereotypes are generally seen to involve being viewed as communal and not agentic, competent, or dominant (Rosette et al., 2016). However, stereotypes related to White women generally overlap highly with those associated with the superordinate category of “women,” and not as much with stereotypes generated for other racialized groups of women(Ghavami and Peplau, 2013; Rosette et al., 2016).

Scholars have posited that the prototype of gender, removed from other social markers, is implicitly racialized as White. This sits within a more general tendency for practitioners and academics to intertwine the gendered category of women with Whiteness (Koenig and Eagly, 2014), due, in part, to the historical exclusion of racially marginalized women from major women’s movements (e.g., suffrage movement; Simons, 1979). This historical exclusion, White supremacy, and racism has led to White women’s experiences to be the center of the gender debate. Moreover, by not acknowledging the role of other social markers on gender, including that of racialization, the dominant culture with which Whiteness is such an aspect, becomes universalized. For example, organizational gender equality initiatives are often spearheaded, and almost exclusively involve White women, while scientific research are often conducted by and on White women (Remedios and Snyder, 2015; Rose-Redwood et al., 2017). Taking the academic field of Psychology as an example, there is growing evidence on how research in Europe and North America are run by, prioritize, and serve White people (e.g., scholarly Psychology publications on race being mostly edited by White editors; Roberts et al., 2020). The result of the prototypicality of White women amounts to a focus in gender research on White women, without considering how differing intersections might result in differences in encountered stereotypes, treatments, or outcomes.

While White women are generally cognitively representative of their gender (Ghavami and Peplau, 2013), racially marginalized women are rendered non-prototypical to their gender, and at times, their racial group too (Purdie-Vaughns and Eibach, 2008; Thomas et al., 2014; Schug et al., 2015). As a result, racially marginalized women are at risk of being intersectionality invisible. For example, White women are more quickly identified as women compared to Black women (Johnson et al., 2012) and participants show the poorest memory in remembering Black women compared to White women or Black men (Sesko and Biernat, 2010). Intersectional invisibility also takes form in the underrepresentation of racially marginalized women, as seen among academic Sociology and Psychology staff (Spalter-Roth and William, 2007; Kohout et al., 2014; Leung and Rainone, 2018).

The intersectional invisibility of racially marginalized women may especially exclude them because their gendered experiences are not the same as those of White women. The double marginalization that racially marginalized women can experience may yield both additive and multiplicative effects of discrimination that White women do not face. People may for instance, negatively stereotype a Black woman as a *woman* (e.g., shy), or as a *Black person* (e.g., lazy). Racially marginalized women’s experiences can thus be gendered and racialized. Additionally, racially marginalized women’s experiences can be racially gendered. For example, “Black women are too aggressive” is not the same as “women are shy” and “Black people are lazy” (Bowleg, 2012; Ghavami and Peplau, 2013). This intersection of race and gender for Black women results in unique stereotypes that are not the sum of racial and gender stereotypes. Additive and multiplicative effects of discrimination are also found in other racialized groups, such as Asian women, who face racialized gender stereotypes (e.g., submissiveness) that may not equate to the sum of gender (e.g., shy) and racial stereotypes (e.g., competent) (Keum et al., 2018).

### Heterogeneity Among Racially Marginalized Women

Intersectional invisibility of racially gendered experiences potentially plays itself out differently for different racially marginalized women. While there is research on the stereotypes that various racially marginalized women face, much of diversity intervention research often only focuses on Black women as a target group – if they look at racially marginalized women at all (e.g., Wilton et al., 2015; Apfelbaum et al., 2016). Yet, it is clear that there is a lot of heterogeneity among racially marginalized women.

As touched upon in the previous section, Black women often encounter stereotypes related to aggression and other high agency perceptions (e.g., strong, dominant). These perceptions are based in the notion that Black women are associated with masculinity more frequently than other racialized groups (Hall et al., 2019). These perceptions are different from the stereotypes that Asian women, for example, face. In our research, we additionally examine what Asian women would require in a diversity interventions, as they are one of the fastest growing racial groups in the United States (Bleiweis, 2021) and Europe (Hillman et al., 2005). Like Black women, Asian women are not prototypical of their gender and experience racial other-ness (Giscombe and Mattis, 2002; Zou and Cheryan, 2017). Unlike Black women, Asian women are stereotyped as relatively low in agency (Ghavami and Peplau, 2013), as hyper-feminine (Mukkamala and Suyemoto, 2018), and as highly competent. While Asian women may experience some benefits from being regarded as highly competent that Black or White women may not experience, they also contend with model-minority stereotypes. Moreover, being associated with docility and lower agency contribute to Asian women’s frequent erasure in discussions about social inequality (Teranishi, 2010; Castro and Collins, 2021; Wong and McCullough, 2021) and lowered visibility in roles requiring assertive behavior.

### Investigating and Incorporating Different Intervention Needs

Considering the reviewed literature, compared to White women, racially marginalized women likely perceive different tools and foci to be beneficial for them in a diversity intervention. In other words, racially marginalized women may possess different intervention needs compared to White women. For example, researchers have already well established that racially marginalized women in STEM fields struggle with different obstacles compared to White women (Reyes, 2011; Alfred et al., 2019); therefore, it is likely that the interventions that are designed to help racially marginalized women advance in their field should be different from those for White women. Yet, just as gender is implicitly racialized as White, diversity interventions for women are also most likely implicitly racialized as White and therefore, may not successfully fulfill the needs that racially marginalized women have. Even in institutions where diversity interventions for women co-exist with diversity interventions for racially marginalized groups, the multiple and intersectional stigmas that racially marginalized women contend with are unlikely to be encapsulated by a unidimensional approach to either gender (in which White women are prototypical and more likely to be targeted) or race (in which men are often prototypical and more likely to be targeted). To our knowledge, researchers have only examined broad classes of diversity interventions so far, while the assessment of the content of diversity interventions that may be particularly important for racially marginalized women is still needed.

Research on diversity ideologies and stereotypes point to some relative differences in intervention needs. To illustrate, Asian women face issues when they are in positions of authority that may be due to stereotypes that they are lower in agency. As a result, Asian women may require agency-enhancing interventions more than White women. The popularization of interventions that counteract stereotypes such as emotionality and submissiveness (e.g., via assertiveness training, confidence-building initiatives, and negotiation workshops) may then target these low agency stereotypes that Asian women face. At the same time, while Black women also encounter difficulties as authority figures at work (Rosette et al., 2018), they also are more likely than women from other racialized groups to be selected for leadership roles requiring demonstrations of agency (Galinsky et al., 2013). As a result, intervention needs based on enhancing agency may have lesser appeal for Black women.

Apart from agency-based intervention needs, there may be other requirements that diverge. For instance, members of racially marginalized groups strongly favor an acknowledgment of their racial and ethnic differences and marginalization in organizations (Gündemir et al., 2019). Moreover, racially marginalized groups respond positively when this acknowledgment is the *status quo* (Arends-Tóth and Van De Vijver, 2003; Wolsko et al., 2006; Ryan et al., 2007). Indeed, racially marginalized women perform worse and anticipate higher risk of discrimination in environments when racial and ethnic differences are not acknowledged (Plaut et al., 2018). Thus, within the context of diversity interventions, acknowledging these intersectional differences among women may be an intervention need that racially marginalized women, contrary to White women, find especially important in a successful intervention.

Moreover, while women generally lack professional and informal networks at work (Fearfull and Kamenou, 2006; Kamenou and Fearfull, 2006), Black women and Asian women may be especially disconnected (e.g., Bell and Nkomo, 2003; Liang and Peters-Hawkins, 2017). Yet, the reasons why racially marginalized women may lack networks may not be addressed in diversity interventions designed for prototypical White women. Black women, for example, face negative stereotypes about their competence and face greater pressure to undergo impression management to be perceived as legitimate (Thomas and Hollenshead, 2001; Bell and Nkomo, 2003; Williams and Dempsey, 2014). As a result, they are less likely to share their non-work identities and engage in informal engagements (Hewlin, 2003, 2009; Phillips et al., 2009) that contribute to making informal contacts. Asian women, on the other hand, have reported a sense of invisibility because of expectations that they are hyper-competent and accomplish their work without challenges (Liang and Peters-Hawkins, 2017). Relatedly, Asian women have been found to rarely seek out mentorship, due to discomfort with approaching others for guidance and thereby, failing to meet the expectations of the model minority myth. The model minority myth suggests that Asians are more successful than any other racially marginalized group because of their supposedly strong values in hard work, perseverance, and belief in meritocracy (Cheng et al., 2017). Issues of embeddedness can especially be exacerbated academic settings where research work can be very autonomous and independent (Ahern, 2019), where there is much competition for resources (Marafioti and Perretti, 2006), and where relocating to new places is common to one’s career trajectory (Richardson, 2009).

### Present Research

The first aim of this research was to examine whether there are indeed racialized differences in intervention needs for women’s diversity interventions. In Study 1 (*n* = 293), we coded participants’ open-ended responses about the aspects of an intervention that would be beneficial for them. In Study 2 (*n* = 489) participants ranked a list of needs derived from Study 1 in order of their own preferences. The second aim of this research was to observe whether the intervention needs relevant to the different groups of racialized women are represented within actual organizations that advocate for women. In Study 3 (*n* = 92) we analyzed organizations’ websites using textual analysis and content coding to examine whether and how the various intervention needs from the previous studies were included.

While we did not base our sample in the social sciences *per se*, we believe that the present research nonetheless contributes to insights that may apply to diversity intervention design in the social sciences and academia at large. Much of the gendered, racialized, and racially gendered barriers found outside of social sciences are very likely mirrored within this field. Moreover, studying biases and social inequity may lead social scientists to believe that gendered and racialized issues occur less frequently within their occupations or institutions (Matias et al., 2021). However, we must be vigilant of possibly falling into a bias blind spot (Pronin et al., 2002; Wang and Jeon, 2020) and engaging in ways that invisibilize these very inequalities (e.g., Bonilla-Silva and Baiocchi, 2001). In the meantime, this study is meant to be taken as a general start to undertake more attention to possible intersectional differences in diversity interventions for women.

## Study 1

In Study 1, participants provided responses to open-ended questions on the components of a successful diversity intervention. From this, we identified any racialized group differences on the expressed needs in women’s diversity interventions (Pre-registration^1^).

### Method

#### Participants

We recruited employed women based in the U.S. who were over 25 years old and heterosexual, reasoning that other stigmatized identities might influence the intersectional experience of gender and race (Bowleg, 2008; Stragà et al., 2020). During recruitment, we deviated from the pre-registration to recruit enough participants to compare racially marginalized women and White women, as well as examine differences within racially marginalized women. Initially, we recruited 300 participants through Amazon Mechanical Turk (MTurk). We extended recruitment of Asian women on Prolific Academic as the initial recruitment did not meet the minimum sample size for this subgroup; additionally, the data quality of Prolific Academic has been evidence to be higher than MTurk (Pe’er et al., 2021)^2^.

In total, we recruited 293 participants (*X*_age_*_=_* 40.67, *SD*_age_ = 10.90). Of the participants, 161 identified as White women, 61 identified as Black women, 40 identified as non-White Latina^3^ women, and 47 as Asian women. We did not pre-register data exclusions; however, after initial data screening, we excluded participants if they (a) did not respond to the open questions, (b) failed the attention check, (c) provided nonsensical answers to the open questions (i.e., responding with illogical words or phrases, “gajgkladjgg”), (d) used the same response for every item across the measures (even reverse-coded), or (e) did not indicate their racial identification; 8 participants were excluded, the results did not significantly differ with exclusions included. Of the participants, 55.3% reported occupying a leadership role in their workplace, with many participants indicating that they worked in management (27.6%), the service industry (17.7%), sales and office (16.4%), and education (13.0%).

#### Procedure

Participants imagined that they were an employee at a fictional company and read a brochure advertising a women’s leadership program (see “Study 1 YesWomen’s leadership intervention” for the brochure and “Study 1_Survey” for the survey set-up in the Supplementary Materials). In line with typical organizational diversity interventions for women, the brochure (1) only showed images of White women, (2) emphasized agency and empowerment [e.g., “Join Natalie White and her team to learn how to assert yourself into a leadership position,” and (3) implied a monolithic experience among women (e.g., “Program objectives: (…) To share the challenges of tackling the typical workplace biases that all women face”]. After reading the brochure, the participants responded to open-ended questions reacting to the intervention. The questions involved asking participants what they found important in a diversity intervention, what they considered to be missing from the intervention presented to them, and the challenges, stereotypes, and experiences they would anticipate as a woman in a leadership position at work. The participants subsequently reported their demographic and occupational information^4^.

#### Codebook Development

We used a qualitative content analysis on participants’ open-ended responses (Elo and Kyngäs, 2008). Prior to receiving the qualitative data, we developed an initial codebook by deductively deriving codes from research on workplace issues and discrimination experienced by oppressed groups. The two coders, both identifying as women and one as a racially marginalized woman, were blind to participants’ racial identity. After the first readthrough of the data, we added inductively derived subcodes to the word dictionary of any concepts that were, at that point, missing.

Thereafter, we did a first round of coding the whole dataset. Due to insufficient reliability and ambiguity of the codes, we revised the codebook twice to more clearly define the coding criteria. From re-reading the responses, we decided to collapse some codes into overarching intervention needs that connected with the participants’ responses and the literature we based our codes on. For example, from the literature we derived separate codes for whether participants would mention their race (*race mentioned*), mention a need for more multicultural diversity (*multicultural*), refer to other stigmatized social groups besides gender (*multiple stigma*), and mention that not all women’s needs or experiences are the same (*not monoliths)*. Through the revisions, because these codes shared a similar thread of addressing heterogeneity within women, we collapsed them under an overarching intervention need of *addressing intersectional differences* within women. The research analyses were hence conducted on the overarching intervention needs, and not on the subcodes. The full list of the original subcodes and how they were grouped into overarching intervention needs can be found in Table 1. After each revision of the codebook, the two coders recoded the whole dataset. If any disagreements arose, the coders discussed them to see if they could be resolved. This process of revision, coding, and discussion repeated until the inter-rater reliability statistics were up to standard (x > 0.90).

**TABLE 1:** Codebook for intervention needs.

Intervention needs	Description	Subcodes	Description
Addressing intersectional differences	Requiring the intervention to acknowledge heterogeneity within gender	Race mentioned	Reference to one’s own race, ethnicity, or status as (racial or ethnic) minority
		Multicultural	Remarks about diversity, especially ethnically, racially, or nationality; remarking how “White” the program is. Expressing how needs or experiences of racially marginalized women are not represented
		Multiple stigma	Referring to other stigmatized or disadvantaged social groups besides gender (e.g., race, age, motherhood)
		Women as monoliths	Expressing that not all women’s needs or experiences are the same. Expressing a more individualized or personalized focus necessary rather than focusing only on gender
Lacking networks	Requiring the intervention to encourage network building, expressing a desire or lack of personal or professional networks	Relatability	Indicating a need for the program coordinators, guest speakers, or participants involved in the intervention to be relatable (e.g., in function, representing one’s experiences, in professional background, or in goals). Wanting relevance to one’s profession or experiences
		Similar networks	Indicating a need for personally or professionally connecting with someone similar or relatable (e.g., similarity through demographics, or occupations)
		Broad networks	Indicating a need for broadly expanding their contacts
Work-life balance	Indication that work-life balance issues are challenging for participants in the workplace (e.g., maintaining familial relations, distinguishing work from other spheres of life) and a want for addressing work-life balance		
Challenges to authority	Challenges or issues that participants experience as a result of their gender or gendered experiences	Pushback (y/n)	Referring to interpersonal, institutional resistance, or challenges in the workplace related to their gender
		Discrimination	Mentioning past experiences or expectations of actions that discriminate based on social group. Expectations or experiences of discrimination that can be expressed in tangible differences
		Respect	Mentioning past experiences or expectations of not being taken seriously, not having authority, or not being able to garner respect
		Low competence	Reference to the individual or women as a group, not being qualified enough or not embodying appropriate leadership characteristics (e.g., “too emotional”)
		Women pushback	Reference to animosity or undermining of authority from women
Insufficient agency perceptions	Describing a need to improve one’s agency, or describing self and others’ perceptions that the participant is not agentic enough	Confidence	Indicating a need to work on one’s confidence (e.g., mentioning that one is too shy or timid). Indicating an importance of assertiveness, confidence, or empowerment training in gender interventions
		Insufficient agency	The perception that the individual, or women as a group are insufficiently agentic to be leaders or successful in the workplace; reference to being too weak or not tough enough
		Excessive agency	Referring to backlash that is faced when the individual or women as a group are behaving in an agentic manner
Excessive agency perceptions	Describing a need to seem less agentic, or describing self and others’ perceptions that the participant is too agentic		

*The overarching intervention needs were aggregated throughout revisions of the codebook. The descriptions of the overarching intervention needs describe what the authors view as the underlying similarities between the subcodes that were originally created from the literature and first readthrough of the participants’ responses.*

We extracted six overarching intervention needs that were collapsed across the responses of the open questions: 42.3% of the participants discussed the importance of *addressing intersectional differences* (e.g., “[The intervention] should also be streamlined for a selected sector of individuals (minorities, gender specific, or sexual orientation)”; “Being a wom[a]n of color I would like this program to also bring up these issues that color women face in today’s workplace and how they can overcome these issues”), 31.1% discussed *improving networks* (e.g., “I think that a leadership program would also offer me a support network”; “One on one mentorship should be a[n] option for those who need it”), and 25.6 discussed *improving work-life balance* (e.g., “[I would like] concrete examples of dealing with family/work conflicts”; “How to manage work and home life would be an awesome topic to review”). When participants discussed issues and challenges in their workplace, 86% mentioned *challenges to authority* (e.g., “I expect to face challenges related to people taking my seriously”; “Male superiors would tend not to take me seriously or give me their full attention”), 47.8% mentioned *addressing perceptions of insufficient agency* (e.g., “[People think] that I am too soft hearted”; “I think some people see women as weaker, or easy to walk over”), and 11.6% mentioned *addressing perceptions of excessive agency perceptions* (e.g., “If we’re too outspoken, we’re bossy”; “And I would not be able to get upset or reprimand someone without being called derogatory names”).

### Results

**FIGURE 1:**
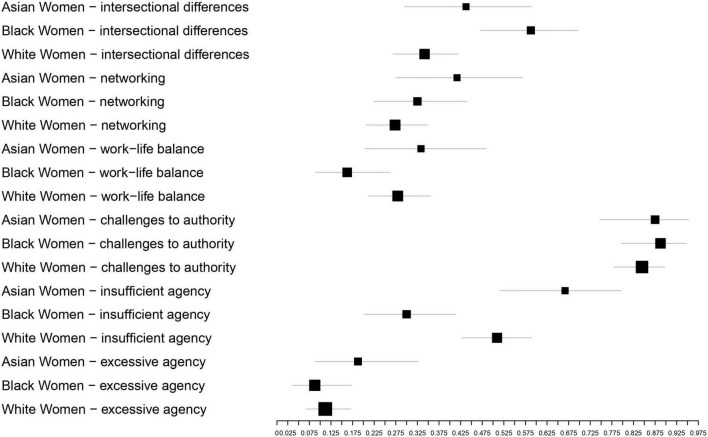
Proportions of intervention needs as a function of racialized group.

Black and Latina women mentioned the importance of incorporating *intersectional differences* more often than White women. Unexpectedly, Asian women did not mention *intersectional differences* more than White women. However, as expected, White women and Asian women mentioned concerns about *insufficient agency* more than Black and Latina women. Asian women also mentioned *improving networks* marginally more frequently than White women. Moreover, Asian women and White women significantly mentioned *improving work-life balance* more than Black and Latina women. The chi-squared statistics and proportions of the intervention needs can be found in Table 2 and Figure 1.

**TABLE 2:** Chi-squared statistics of intervention needs per racialized group.

	Frequency	*p̂* Asian Women	*p̂* Black/Latina Women	*p̂* White Women	Asian:Black/Latina	Asian:White	Black/Latina:White
Addressing intersectional differences	124	0.438	0.588	0.342	χ2 = 2.711	χ2 = 1.469	χ2 = 13.237
					*p* = 0.100	*p* = 0.225	*p* < 0.001
Improving networks	91	0.417	0.325	0.273	χ2 = 1.095	χ2 = 3.578	χ2 = 0.684
					*p* = 0.295	*p* = 0.059	*p* = 0.408
Improving work-life balance	75	0.333	0.163	0.280	χ2 = 4.996	χ2 = 0.518	χ2 = 4.004
					*p* = 0.025	*p* = 0.472	*p* = 0.045
Challenges to authority	252	0.888	0.888	0.845	χ2 = 0.045	χ2 = 0.268	χ2 = 1.119
					*p* = 0.831	*p* = 0.604	*p* = 0.290
Addressing perceptions of insufficient agency	140	0.667	0.300	0.509	χ2 = 16.389	χ2 = 3.692	χ2 = 9.504
					*p* < 0.001	*p* = 0.055	*p* = 0.002
Addressing perceptions of excessive agency	34	0.188	0.088	0.112	χ2 = 2.743	χ2 = 1.883	χ2 = 0.335
					*p* = 0.098	*p* < 0.170	*p* = 0.563
							

### Study 1 Discussion

The aim of this study was to investigate any differences in intervention needs among different racialized groups of women. Aligning with agency stereotypes faced by each group, we found that White women and Asian women more frequently mentioned a need to address perceptions of insufficient agency more than Black and Latina women. Contrary to our expectations, only Black and Latina women notably mentioned incorporating intersectional considerations in diversity interventions for women more frequently than White women. There could be various explanations for why no relative differences were found with Asian women. First, the relatively low sample size may not be representative of the broad variability in experiences present among Asian women. Second, Asians have been shown to encounter great pressure to assimilate into Eurocentric notions of success and consequently downplay their racial and ethnic differences (Dennis, 2018). This may have contributed to fewer Asian women willing to emphasize their racial or ethnic differences. Before speculating further, however, we wanted to see if this effect would replicate in a follow-up study (i.e., Study 2).

Additionally, our findings that Asian women more frequently responded with a need to improve their networks aligns with research showing Asian women’s reported sense of invisibility in the workplace (e.g., Liang and Peters-Hawkins, 2017). These findings may suggest that at least when asked to self-report, Asian women perceived a lack of embeddedness and resources to build networks that are favorable for them.

Lastly, our findings for the work-life balance intervention need showed that Asian and White women mentioned improving work-life balance more than Black women. Despite not being the only caregiving responsibility that women disproportionately bear at home, motherhood can be deeply intertwined with balancing one’s work and private life. A possible explanation for our results may be that Black and Latina women may be relatively more hesitant to emphasize notions of motherhood or work-life balance due to being associated with negative stereotypes as bad mothers (e.g., welfare queen stereotype for Black women; Rosenthal and Lobel, 2016) or hyper-fertile (e.g., “breeders” stereotype for Latina women; Gutiérrez, 2009). In comparison, White women and Asian women may face these kinds of associations less frequently. These results do not indicate that that work-life balance resources are more or less relevant for any particular racialized group. However, our rationale does suggest that racially gendered stereotypes may not only affect one’s preferences for an intervention, but they may also affect the willingness to express these preferences and be associated with particular interventions.

## Study 2

In Study 2, we examined how women ranked the importance of the intervention needs identified in Study 1. To address the limitation of having participants react to a particular diversity program in Study 1 and solely a U.S.-based sample, we conducted study 2 to gauge participants’ prioritization of intervention needs more generally. In Study 1, we saw the biggest differences in how often *intersectional differences* and perceptions of *insufficient agency* were discussed. Therefore, in Study 2, we anticipated a similar pattern of results where *intersectional differences* and *insufficient agency* would show the biggest ranking differences – we hypothesized:

H1: Black women would prioritize consideration for *intersectional considerations* more than White women.
H2: White women and Asian women would prioritize addressing *perceptions of insufficient agency* more than Black women.

While we aggregated Latina and Black women as we assumed that these groups encounter similar stereotypes (Fiske et al., 2002) in Study 1, we decided not to do so in Study 2 because they each also face unique marginalization that we did not account for Zou and Cheryan (2017).^5^ Therefore, only Asian, White, and Black women participated in this study.

### Participants

We conducted the survey via Prolific Academic and removed participants if they (a) did not engage in the ranking task, or (b) if their other responses indicated that they had not taken the questionnaire seriously by indicating the same scores for every item (even reverse-coded). The final sample consisted of 489 women, with 302 White women (61.8%), 98 Black women (20.0%), and 89 Asian women (18.2%). The mean age of the sample was 27.20 (*SD* = 7.97). Of participants, 34.6% of the participants indicated having had experience in a leadership position in their workplace. Most of the sample resided in the United Kingdom (55.2%), and the United States (30.3%), with the remainder in Europe (11.8%), and Canada or Australia (2.7% combined).

### Procedure

Participants imagined that their workplace had invited them to participate in a diversity intervention for women and ranked items for potential inclusion in the intervention by personal order of importance (see “Study 2_Survey” for the survey set-up in Supplementary Materials). The original survey included 12 items that participants could rank, but we only included six items that most resembled the intervention needs categorized in Study 1. Rankings were reverse-coded, where higher scores indicated a higher prioritization. The rankings included in the main analyses were: *addressing intersectional differences* (“addressing how race influences gender in the workplace”), *improving networks* (“networking opportunities”), *improving work-life balance [“*Discussing how to deal with work-life balance (i.e., parental or other personal issues*)”]*, *addressing challenges to one’s authority* [“Dealing with push-back or stereotypes in your workplace (e.g., coping with conflicting expectations, assumptions of incompetence, challenges to authority)”], *dealing with perceptions of insufficient agency* (“addressing the belief that women are not assertive”), and *dealing with perceptions of excessive agency* (“Addressing the held belief that assertive women are too bossy or dominant”). After ranking, the participants completed several measures^6^ and indicated their demographics.

Each ranked item was treated as an ordinal variable in the analyses. For each intervention need we used Kruskal–Wallis tests to identify significant group differences between any of the racialized groups. Once a significant difference was found, we further looked at the breakdown of differences between Asian women, Black, women, and White women to identify the significant contrasts; for this, we used Dunn’s *post hoc* test.

### Results

The results for the Kruskal–Wallis and *post hoc* Dunn’s tests are found in Tables 3, 4.

**TABLE 3:** Differences in rankings of the intervention needs between racialized group.

	Addressing intersectional differences	Improving networks	Improving work-life balance	Addressing challenges to authority	Dealing with perceptions of insufficient agency	Dealing with perceptions of excessive agency
Kruskal–Wallis *H*	29.624	2.346	0.816	5.8541	10.519	6.162
df	2	2	2	2	2	2
Asymp. Sig.	<0.001	0.310	0.665	0.0536	<0.001	0.046

**TABLE 4:** Mean rankings, standard deviations (in parentheses), and Dunn tests’ contrasts of each intervention need per racialized group.

Intervention needs	Asian women	Black women	White women	Comparison	*z*	*p*. adj
Intersectional differences	3.989 (1.578)	4.796 (1.324)	3.666 (1.824)	Asian:Black	–3.348	<0.001
				Asian:White	1.181	<0.001
				Black:White	5.442	<0.001
Networking	3.798 (1.866)	3.408 (1.793)	3.513 (1.770)	Asian:Black	1.470	0.424
				Asian:White	1.266	0.308
				Black:White	–0.538	0.590
Work-life balance	3.034 (1.715)	2.908 (1.650)	3.096 (1.719)	Asian:Black	0.436	0.994
				Asian:White	–0.332	0.740
				Black:White	–0.895	1.000
Challenges to authority	3.753 (1.805)	3.133 (1.791)	3.364 (1.744)	Asian:Black	2.395	0.050
				Asian:White	1.791	0.110
				Black:White	–1.158	0.247
Insufficient agency	3.303 (1.465)	3.306 (1.509)	3.765 (1.519)	Asian:Black	–0.015	0.988
				Asian:White	–2.511	0.018
				Black:White	–2.587	0.029
Excessive agency	3.124 (1.608)	3.449 (1.507)	3.596 (1.581)	Asian:Black	–1.359	0.261
				Asian:White	–2.467	0.041
				Black:White	–0.848	0.396

Overall, the standard deviations for each intervention need suggests high variation within each racialized group for how each intervention need was ranked. Therefore, it must be borne in mind that any group differences found may be on the aggregate level, but individual participants may differ widely in how they provided rankings. Consistent with Study 1, Black women ranked *intersectional differences* significantly higher than White women. Contrary to Study 1, however, Asian women also ranked *intersectional differences* significantly higher than White women. Moreover, as in Study 1, White women ranked *dealing with perceptions of insufficient agency* higher than Black women, though the difference between Asian and Black women was not statistically significant. At the same time, interestingly, White women ranked *dealing with perceptions of excessive agency* higher than Asian women. White women and Asian women ranked *challenges to authority* significantly higher than Black women. Unlike in Study 1, there were no significant differences in the rankings for *work-life balance* or *networking* among any of the racialized groups of women.

### Study 2 Discussion

When looking at how women prioritized intervention needs differently in a diversity intervention for women, our first hypothesis was that Black women may value interventions that addressed their racialization alongside their gender. This was supported. Unlike in Study 1, with a larger Asian sample and presumably more statistical power, we *were* able to find that Asian women prioritized incorporating intersectional differences higher than White women. This result provides some evidence that Black women and Asian women may require an acknowledgment of how race affects their gendered experiences compared to White women due to their racial marginalization.

In our second hypothesis we anticipated that White women and Asian women would prioritize interventions that addressed insufficient agency more than Black women. Consistent with Study 1, White women ranked interventions that addressed perceptions of insufficient agency higher than Black women. Surprisingly, White women also ranked addressing perceptions excessive agency higher than Asian women. This finding may suggest that White women may be more concerned with balancing perceptions of agency than Black and Asian women.

Inconsistent with Study 1, Asian women did not differ significantly from Black women on the importance of addressing perceptions on insufficient agency. This inconsistency may firstly be connected to the immense variability in racialized experiences and stereotypes that Asian woman are confronted with, both within and across different Asian communities. This may be especially so as the first study was U.S.-based, and the second study sampled a broader participant pool. In Study 2, a higher South and South-East Asian population was represented compared to Study 1 (Study 1 = 38.5%, Study 2 = 46.0%), where a higher East Asian population participated. Research has shown a lot of variation in how different groups of Asian women encounter different agency-related stereotypes, for example, showing that some South Asians (e.g., Bangladeshis or Pakistanis) are perceived to be more assertive compared to some East Asians (e.g., Vietnamese or Koreans) (Ramakrishnan et al., 2017; Hassan, 2018). Arguably, the differences in the intervention needs that would accurately reflect the intra-group diversity among Asians are more pronounced than what is currently presented.

Secondly, we theorize that the discrepancy in results between these first two studies may be based on differences in self versus other perceptions. That is, the perceptions of a group may differ based on whether someone is a member of that group (i.e., self-perceptions) or outside of the group (i.e., other perceptions). Research with Asian women have detailed a discrepancy in how agentic they view themselves from the perceptions that others have of them (Cheryan and Markus, 2020). Even though Asian women are stereotyped by others to be closer to traditional femininity and report feeling pressure to behave accordingly (Williams et al., 2016), Asian women have rated themselves as more assertive than White women (Toosi et al., 2019). With that logic, Study 1 may have been more conducive for participants to think about others’ perceptions, because we asked them to think about how others’ stereotypes and treatment of them would elicit intervention needs. In turn, Study 2 may have been more conducive for participants to think about self-perceptions because we asked them to order the intervention needs based on their personal needs. Therefore, while Asian participants may bring up addressing perceptions of insufficient agency because that is how others view them, they may not prioritize this intervention need as they may not see themselves as actually lacking in agency.

Even though no significant differences were found in Study 1 for challenges to authority, in this study, Asian and White women ranked tackling challenges to authority significantly higher than Black women. Challenges to authority and insufficient agency may, in hindsight, tap into similar theoretical issues, such that the stereotypes for Black women are more similar to stereotypes of men, and that Asian women and White women are perceived as relatively less assertive and assured (Ghavami and Peplau, 2013; Rosette et al., 2016; Rosette et al., 2018; Hall et al., 2019). In fact, tackling challenges to authority may be more reflective of the stereotypes that others have of Asian women than their own sense of agency. Having ranked challenges to authority higher may also explain why the rankings for insufficient agency are relatively lower for Asian women, when compared to the other racialized groups.

Lastly, our results indicate that there were also shared intervention needs across the racialized groups in this study. For instance, improving networking or work-life balance were ranked relatively similarly; these results could indicate that when made to choose between other intervention needs (and not self-generate as in Study 1), participants are similarly in need of work-life balance and networking elements in a diversity intervention for women.

## Study 3

In our final study, we examined the extent to which the intervention needs identified in previous studies were recognized and addressed by organizations. We scraped and analyzed the public websites of companies that pledged to promote women’s representation. In line with intersectional invisibility research, we expected that the intervention needs that were more relevant to multiply marginalized groups would be less represented among organizations than the intervention needs that were more relevant to White women as singly marginalized groups (Purdie-Vaughns and Eibach, 2008; Thomas et al., 2014). Specifically, we anticipated that agency-enhancing needs that seemed to be more relevant for White women would be represented the most among the organizations.

### Method

#### Sample

The public websites of 186 signatory organizations of the Dutch “Talent naar de Top” (ENG: Talent to the Top; TndT) diversity charter were used. The charter allows private enterprises and public organizations to publicly commit to promoting women’s representation in top management positions (Talent naar de Top, 2020). We only mined websites that advocated or referred to women, including efforts to promote women, foster women’s inclusion, or inform the public about their interventions for women. Signatories without any website information advocating for women were not included, resulting in a final sample of 92 organizations.

#### Procedure

After screening and scraping organizational websites, we compiled a word dictionary based on the intervention needs identified in Study 1 using procedure. In this procedure, prior to engaging with the websites, the first author and a second expert outside of the project used Weber’s (2005) procedure and generated a literature-based version of the word dictionary for each intervention need. Words were generated for each intervention need based on the responses to the open-ended questions from Study 1 and related diversity research. For instance, we drew on Pietraszkiewicz et al. (2019) agency word dictionary by borrowing from their agency words and adding our own words based on the quotes that were coded under the agency categories in Study 1. A similar procedure was used for the other intervention needs of the word dictionary. Following the creation of these preliminary lists, the coders brainstormed to generate other relevant words for each category and added them to the initial lists. This process mainly involved generating synonyms of adjectives (e.g., shy, timid). Additionally, the coders attempted to streamline the lists as much as possible by including the word stems of relevant words (e.g., including empower* in the word list that would accept all words that start with “empower-,” rather than including “empower,” “empowerment,” “empowering” as separate entries).

Subsequently, the two coders each independently coded 10% of the sample to review, revise, and check for the saturation of the word dictionary (i.e., the point at which no additional words could be contributed to the word dictionary). From this, we arrived at a preliminary word dictionary. Because many websites were only in Dutch, we translated the dictionary from English to Dutch (Singh et al., 2006) through joint discussions with native Dutch speakers external to the project. At this stage, we also added variations of adjectives for proper nouns that are used in Dutch, depending on whether the adjective describes a noun with a “de” or “het” article in Dutch. An example of this is the word “Chinees” in Dutch (ENG: Chinese), which can be used as a proper noun or an adjective for a “het” noun. Other variations of “Chinees” are “Chinese,” the adjective used for “de” nouns, and “Chinezen,” the plural form of the proper noun.

We then conducted a post-measurement validation to fine-tune the word dictionary. Through an iterative procedure of human and LIWC computer coding (Weber, 2005), we first manually coded a subset (10%) of the documents using the preliminary word dictionary. Then we ran these documents through the LIWC program. Together, we calculated a “hit rate” and “false hit rate”; if the hit rate was less than 80 and the false hit rate was more than 10%, revisions would be made to the word dictionary. This process was continued for five iterations until the hit rates and false hit rates were satisfactory across all categories (see “Study 3_Establishing Word Dictionary Reliability” in the Supplementary Materials), arriving to the final version of the word dictionary (see “Study 3_Word dictionary” in the Supplementary Materials). Using the finalized word dictionary, we used the Linguistic Inquiry and Word Count program (LIWC) to scan the websites.

The categories in the word dictionary largely coincided with their corresponding intervention needs found in Study 1; however, “challenges to authority” was not included because these specific individual experiences could not be detected using LIWC and may have been confounded with the agency category. Ultimately, we used six categories: *agency*, *insufficient agency, excessive agency, intersectional differences*, *networking, and work-life balance*. *Insufficient agency* (e.g., “docile,” “shy”) and *excessive agency* (e.g., “bossy,” “aggressive”) related to being perceived as too agentic, or not agentic enough. Compared to intervention needs found in Study 1, we added a general referral to *agency* as a category (e.g., “assertiveness,” “confidence”) to account for related words that do not carry as much valence as *insufficient* and *excessive agency*. *Intersectional differences* included words or phrases associated with multicultural representation, racial or ethnic representation, and reference to stigmatized groups and identities other than women (e.g., “cultural background,” “skin color”). Because we examined these other stigmatized identities within the organizations’ advocacy of women, this coding proxied an acknowledgment of differences within women. Moreover, to check for possible over-estimation of this category, we used the quanteda package in R to see whether race and ethnicity-related word co-occurred within a window of five words (pre- and post-) with “women” throughout the texts. *Networking* consisted of words and phrases related to role-models, references to community, or expanding the professional and personal contacts of women (e.g., “connection,” “mentor*”). *Work-life balance* included words related to negotiating ones’ career and caregiving responsibilities, or one’s general personal life (e.g., “flexible,” “work-life”).

The LIWC provided crude percentages based on the frequency with which a word or short phrase had been detected within our word dictionary, relative to the total number of words in the text. We calculated the prevalence and their respective ranges of each category across the organizations wholly (see Table 5) and split by industry (see Table 6). Splitting the data by industry was done to gain insight in how the intervention needs were represented in the Education, Science, and Research sector, where social science research is most likely to take place. Moreover, we calculated the total percentage of organizations that mentioned each category in any capacity (i.e., more than 0% prevalence in the categories). Lastly, we used chi-squared analyses to determine if any category was significantly associated with each other (see Table 7). In addition to reporting the LIWC results in the next section, we footnoted supporting quotes and remarks that we made during the human coding that reflected more conceptual content to support the LIWC results.

**TABLE 5:** Prevalence of intervention needs.

Category	Prevalence (%)	Range (%)	Organizations with > 0 prevalence (%)
Agency	2.010	6.120	97.8
Insufficient agency	0.010	0.090	9.7
Excessive agency	0.010	0.240	18.3
Intersectional differences	0.090	1.220	51.6
Networking	0.660	6.800	90.3
Work-life balance	0.390	1.060	55.9

**TABLE 6:** Prevalence of intervention needs per industry.

Category	Prevalence (%)	Range (%)	Organizations with > 0 prevalence (%)
**Accountancy, Banking, and Finance (*N* = 11)**			
Agency	2.477	2.630	100.0
Insufficient agency	0.000	0.000	0.0
Excessive agency	0.000	0.000	0.0
Intersectional differences	0.073	0.370	54.5
Networking	1.319	6.610	100.0
Work-life balance	0.077	0.440	27.3
**Business, Consulting, and Management (*N* = 19)**			
Agency	2.248	5.330	94.7
Insufficient agency	0.006	0.090	15.8
Excessive agency	0.020	0.240	42.1
Intersectional differences	0.107	0.480	73.7
Networking	0.746	2.400	89.5
Work-life balance	0.247	0.850	78.9
**Education, Science, and Research (*N* = 18)**			
Agency	1.731	3.960	94.4
Insufficient agency	0.003	0.060	5.6
Excessive agency	0.006	0.060	16.7
Intersectional differences	0.167	1.220	44.4
Networking	1.194	4.710	94.4
Work-life balance	0.330	0.330	44.4
**Information Technology (*N* = 4)**			
Agency	2.230	1.630	100.0
Insufficient agency	0.000	0.000	8.3
Excessive agency	0.003	0.010	25.0
Intersectional differences	0.053	0.090	75.0
Networking	0.630	1.120	75.0
Work-life balance	0.155	0.300	100.0
**Law (*N* = 12)**			
Agency	1.613	2.950	100.0
Insufficient agency	0.008	0.010	28.6
Excessive agency	0.006	0.030	25.0
Intersectional differences	0.041	0.250	41.7
Networking	0.833	2.260	100.0
Work-life balance	0.161	0.650	50.0
**Property, Manufacturing, and Construction (*N* = 7)**			
Agency	1.514	0.790	100.0
Insufficient agency	0.010	0.040	28.6
Excessive agency	0.001	0.010	14.3
Intersectional differences	0.133	0.530	71.4
Networking	0.537	1.440	85.7
Work-life balance	0.250	1.060	85.7
**Public Services and Administration (*N* = 6)**			
Agency	3.385	5.260	100.0
Insufficient agency	0.000	0.000	0.0
Excessive agency	0.000	0.000	0.0
Intersectional differences	0.272	1.170	33.3
Networking	0.587	2.040	66.7
Work-life balance	0.000	0.000	0.0
**Retail and Services (*N* = 15)**			
Agency	2.279	3.350	100.0
Insufficient agency	0.006	0.090	6.7
Excessive agency	0.000	0.000	0.0
Intersectional differences	0.050	0.420	26.7
Networking	1.358	3.650	86.7
Work-life balance	0.145	0.560	60.0

**TABLE 7:** Chi-squared statistics of intervention needs.

Comparisons		
Agency	Intersectional Differences	χ*2* = 2.180
		*p* = 0.140
	Networking	χ*2* = 19.077
		*p* < 0.001
	Work-Life Balance	χ*2* = 2.592
		*p* = 0.107
Intersectional Differences	Insufficient Agency	χ*2* = 0.019
		*p* = 0.019
	Excessive Agency	χ*2* = 15.049
		*p* < 0.001
	Networking	χ*2* = 6.545
		*p* = 0.011
	Work-Life Balance	χ*2* = 14.660
		*p* < 0.001
Networking	Insufficient Agency	χ*2* = 1.068
		*p* = 0.301
	Excessive Agency	χ*2* = 2.229
		*p* = 0.135
	Work-Life Balance	χ*2* = 2.061
		*p* = 0.151
Work-Life Balance	Insufficient Agency	χ*2* = 7.856
		*p* = 0.005
	Excessive Agency	χ*2* = 12.317
		*p* < 0.001

### Results

Notably, only about 49% of the companies that were members of TndT showed some content of their advocacy for women on their websites. As expected, *agency* was the intervention need most represented out according to the LIWC results and manual coding with 97.8% of the organizations representing *agency* in some way in their websites. When disaggregating by industry, “we still observed that at least 90% of organizations mentioned agency across all industries.” However, words related to *insufficient agency* and *excessive agency* were mentioned the least across the organizations.^7^ *Networking* was the second most frequent category. The prevalence of networking words was mentioned by 90.3% across all organizations, and by more than 85% of organizations in each industry. The chi-squared analyses also showed that *agency* and *networking* words were significantly associated with each other.^8^

Words associated with *work-life balance* was third most prevalent amongst the intervention needs, after *agency* and *networking*, and was mentioned by 55.9% of the organizations. Compared to *agency* and *networking*, there was more variation in how individual sectors represented *work-life balance*. The sectors with which *work-life balance* was mostly represented were Information Technology (100% across all organizations) and Property, Manufacturing, and Construction (85.7% across all organizations). The sectors with which *work-life balance* was least represented in the organizations’ websites were in Public Services and Administration (0% across all organizations) and Accountancy, Banking, and Finance (27.3% across all organizations).

Lastly, while *intersectional differences* was indicated by the LIWC to be mentioned by a little over than a half of the organizations, the prevalence of words related to this category was relatively low. Moreover, the co-occurrence analysis suggests that the websites may have mentioned other social groups besides gender in their advocacy of women, but mainly as separate groups. The organizations seemed to rarely refer other social groups’ intersection with women as “women” co-occurred with race and ethnicity related words only 27 times out of a corpus of approximately 36,000 words.^9^

When zooming in on the Education, Science, and Research sector, the patterns of the data parallel that of looking at all the organizations. *Agency* and *networking* were most represented among the intervention needs, and addressing perceptions of *insufficient agency* and *excessive agency* were the least represented. While *intersectional differences* and *work-life balance* seemed to be represented equally when considering whether the organizations represented these needs at all, the prevalence of words related *intersectional difference* was still roughly one-half that of *work-life balance*.

### Study 3 Discussion

The aim of Study 3 was to examine the extent to which the intervention needs of various racialized groups of women were represented in a sample of organizations that advocated for women. Only roughly half of the signatories had any website relating to the advocacy and promotion of women. Of those organizations, agency, networking, and work-life balance were prominent intervention needs. However, showcasing agency was the most prominent as it was also referenced in conjunction with other intervention needs. Few organizations mentioned perceptions of excessive agency that may affect women at the workplace. Representing the need to boost one’s agency to be successful while failing to emphasize the potential consequences of being perceived as excessively agentic reinforces the White prototype that women’s issues exclusively concern perceptions of insufficient agency. This approach poses a particular risk of excluding Black women, where addressing perceptions of insufficient agency was less relevant for them.

The prevalence of organizations acknowledging intersectional differences seems low, particularly when considering an actual intersection of gender and other social groups. While stigmatized groups other than gender seemed to be named frequently by organizations, in the co-occurrence analysis we saw that they were often discussed as independent entities rather than intersectionally. It is, therefore, not definitive that organizations, including the Education, Science, and Research sector, are strongly articulating intersectional considerations. Lacking specificity when discussing how different social intersections differentially impact women’s experiences may be a notable shortcoming, particularly for Black women and Asian women who highly prioritize addressing intersectional differences in a diversity intervention for women.

## General Discussion

In this research, we found evidence of differences between different racialized groups of women in their intervention needs for a successful diversity intervention for women. We found that White women and Asian women prioritized addressing perceptions of insufficient agency more than Black women across our first two studies. These findings fit with the stereotypes that each group faces, where Black women are not generally perceived to be lacking in agency, while White women and Asian women are (Rosette et al., 2016, 2018). Our finding in Study 3 that organizations widely promoted enhancing agency perceptions may, therefore, be more likely to serve the intervention needs for Asian and White women, particularly if they involve dealing with others’ perceptions of these women and challenges to their authority.

However, the organizations in Study 3 rarely mentioned perceptions of excessive agency in their advocacy of women. Excluding this element may neglect the intervention needs of White women who may be concerned with balancing insufficient and excessive agency perceptions, as shown in Study 2’s findings where White women ranked addressing both agency perceptions relatively highly. Additionally, organizations who fail to address excessive agency may also exclude Black women as they are more likely than Asian or White women to be prescribed as more dominant and even aggressive (Rosette et al., 2016). Therefore, diversity interventions that seek to use perceptions of agency as a point of training for women may benefit from incorporating how perceptions of excessive agency affect women, and the idea of balancing on the tightrope between being perceived as too agentic and insufficiently agentic.

Across the first two studies, Black women consistently brought up and prioritized intersectional differences more than White women; Asian women also showed this pattern in the second study. When considering this prioritization with the results of Study 3, we saw that while other stigmatized identities besides gender were recognized among the organizations, the explicit link to these marginalizations and gender were often unmentioned and unexplored. Therefore, our findings in Study 3 that the organizations did not conclusively address intersectional considerations present another source of exclusion of intervention needs that may be especially relevant for Black women and Asian women.

The exclusion of intersectional considerations overlap with other intervention needs too. Although we studied incorporating intersectional differences as a separate intervention need, intersectional considerations theoretically extend to other intervention needs. Despite finding a sizeable representation of work-life balance policies and networking in our sample of organizations in the third study, the lack of an intersectional consideration may compromise these needs for racially marginalized women, too.

To illustrate, in the second study we found that the three groups of participants shared a concern for work-life balance and networking as intervention needs. Yet, different racially gendered obstacles may fuel this prioritization for these two needs. It may be possible that a networking intervention for Asian women requires an understanding of the invisibility that can often be felt by Asians, and the model-minority myth that can act as a barrier for Asian women to seek out mentoring and professional help. This reasoning for having problems with networking is different from the issue of networking for Black women who may be more likely to lack informal connections and may need wrestle with impression management in their workspaces.

In the same vein, lacking intersectional considerations when addressing work-life balance aspects of a diversity intervention miss out on possible important nuances for different groups’ motivations. As theorized in Study 2, even though work-life balance policies may be very important for Black women, they may be hesitant to express and associate themselves with the negative stereotypes that are associated with Black women and motherhood. Organizations and institutions therefore must also be able to recognize the different racialized struggles that these women face to be able to adequately help them navigate through these struggles, participate in diversity interventions, and comprehensively benefit from them.

### Implications for Academic Social Sciences

Our findings have implication for the social sciences, and academia more broadly. After all, in the third study we saw that the Education, Science and Research sector is not impervious to showcasing agency while being limited in their intersectional scope. Therefore, the findings in this research are likely to emerge in their own form in the academic social sciences.

For instance, as in any job position, work in academia involve evaluations. Evaluations are present for teaching, research performance, when applying for grants, and when considering tenure should be granted. There is already much evidence showing that evaluations within academia are skewed negatively toward women (Llorens et al., 2021). However, as the first and second study show, it is incomplete to assume that these biases are one and the same for all women. That is, differential perceptions of insufficient agency and excessive agency are undoubtedly present, and result in different trajectories for the same possible negative evaluations. Being aware of how these biases differ may be relevant for academic and research institutions to take the appropriate steps to mitigate any respective negative consequences.

Additionally, to increase women’s representation in academia, many universities are increasingly establishing diversity interventions for women throughout their own staffing and retention procedures (e.g., via targets or quotas; European Commission, 2019). However, without specifically attuning to intersectional differences, such initiatives are at risk of— through the prototypicality of Whiteness and atypicality of racially marginalized women— exclusively divesting efforts toward the benefit of White and female scholars. As a result, women from racially marginalized groups are further overlooked. Furthermore, by having interventions that seemingly target all women, such exclusive interventions may contribute to the further legitimization of inequality of racially marginalized women in these spaces (Brady et al., 2015).

### Limitations

We based our research focus and findings on the context of Western Europe and North America. Consequently, we examined racialized differences between Asian women, Black women, and White women because these three marginalized groups are the best understood in the stereotyping literature in these contexts. However, the openness to which racially marginalized groups are expressive and considerate of racialized or ethnic differences relative to White majority groups is different from contexts where race and ethnicity discourse is less present (e.g., Law and Zakharov, 2019). Moreover, one can surmise that because Western Europe and North American both have colonial and imperial histories, that this is linked to many racially marginalized people leaving their home countries as economic and educational migrants. Racially marginalized people within these contexts may therefore be more likely to participate in the labor market in professional and service industries where diversity interventions are implemented in the first place. The importance of racialized and ethnic differences, and the resources to tend to these differences, may differ from places such as Latin America and the Caribbean where racial dynamics are very different from North America and Western Europe (England, 2010; Golash-Boza and Bonilla-Silva, 2013), and where similar diversity interventions are less likely to be present.

Moreover, White women, Black women, and Asian women do not represent all the ways that gendered experiences can be racialized. Very different ethnicities were combined into racialized categories (e.g., Afro-Caribbean Black women and African-American Black women, or Chinese Asian women and Indian Asian women). Moreover, while we attempted to control for sexual orientation by sampling only heterosexual women, our research is limited in that our theorizations and findings do not consider *trans* and non-binary overlaps of gender with identifying as a woman. Such an approach misses rich heterogeneity in experience between and within racialized and gender groups (Mohr and Purdie-Vaughns, 2015).

Furthermore, not all the intervention needs found in the first two studies may be generalizable to other racialized groups of women who were not included in this research. Middle-Eastern and North African women for example, are likelier than Asian or Black women to be perceived as Muslim and struggle with stereotypes of being “repressed,” while at the same time maneuvering stereotypes of excessive agency as “Angry Arabs” (Hamad, 2020). Indigenous or aboriginal women in settler colonial contexts may have intervention needs that are more likely to be informed by navigating through competence stereotypes that they are uneducated and undisciplined, yet spiritual and wise (Morrison et al., 2008). These varied and somewhat contrasting experiences may then also translate to other intervention needs and their respective prioritizations that were not included in this research. At the same time, while the exact constellation of intervention needs may not exactly map on to other contexts and racializations, the pattern that intervention needs differ among women based on racialization likely extends to other racially marginalized groups.

### Future Directions

The findings of our studies may lead to many future avenues of research on intervention needs for racially marginalized women. After providing evidence that intervention needs may differ based on racialization, empirically connecting these differences with research on racially gendered experiences and stereotypes would provide further insights on how organizations can precisely nuance and improve their interventions. For instance, if the pattern of results for Asian women are indeed connected to the model-minority stereotypes that they face, how can this be incorporated in an intervention to improve intervention success? How can what is known about negative competence stereotypes that Black women face be added to diversity interventions to provide Black women with greater access to informal networks? Looking at these finer grained explanations and connecting them to participants’ intervention preferences would offer more content-specific and practical insights into successful diversity intervention design.

Across the first two studies we observed general differences by considering how racialization is intertwined with gender among these intervention preferences. This was, however, only a glimpse of how these differences emerge considering various intersectional axes. Research on diversity approaches in organizations, for instance, show that numerical representation of racially marginalized folks affects their preferences in approaches (Apfelbaum et al., 2016). While we could not show this with our data, other factors such as social economic class, education level, and job industry are closely associated with racial representation. Racial minorities are, for instance, often disproportionately represented in lower paying industries (Kmec, 2003; Byrne et al., 2005) that require lower to no formal education. In these contexts where racially marginalized people are at least moderately represented, racially marginalized women may face less representation concerns and may prioritize intersectional differences less and other intervention needs more. Rather, women may prioritize more resource-based interventions, such as work-life balance interventions that may make time as a resource more readily available. On the other hand, scarcity in racial representation, as can be seen in higher educated industries such as the social sciences (Spalter-Roth and William, 2007; Kohout et al., 2014), may place an even greater emphasis on having intersectional differences acknowledged for a racially marginalized member. While no single study can be fully comprehensive of all these factors, it is important to realize in continued research and theorization that intersectional differences among women regarding their intervention needs is sophisticated and can be interpreted in conjunction with many other seemingly unrelated intervention needs.

## Conclusion

It is crucial when designing gender diversity interventions to understand that women are not a monolith. We observed how different Asian, Black, and White women were associated with different intervention needs that aligned with their respective racially gendered stereotypes. Moreover, we found that when organizations discuss their diversity and inclusion efforts for women, they mainly focused on intervention needs associated with enhancing agency. While this focus may fulfill some of the intervention needs for White women and some Asian women, an exclusive focus on agency runs the risk of failing to meet other important intervention needs that these women possess. Black women and Asian women, both of whom prioritized addressing intersectional considerations, are additionally at risk for being excluded from these diversity interventions for women. Moreover, while some intervention needs may be shared by different racialized groups, the rationales for these needs may by racially gendered, and therefore racially gendered intersectional considerations may still be required in these shared concerns.

The range of intervention needs that are required for these diversity interventions suggest that focusing on any one given intervention need is insufficient, and the continued unfulfillment of intervention needs of specific groups of women might ironically exacerbate inequalities. Our results have implications for the social sciences in academia, that is growingly internationalized and that seeks to design their work more and more equitably for racially marginalized groups. Practitioners may likely benefit more from their own local investigation of the intervention needs required in a given group to flexibly design interventions that seek to fulfill participants’ prioritized intervention needs. Otherwise, interested participants with needs other than enhancing agency may be unaffected by these diversity interventions for women that seemingly help them, or feel actively excluded. Both of which will ultimately negatively affect racially marginalized women’s inclusion and put them at a greater disadvantage in an already competitive environment.

## Data Availability Statement

The datasets presented in this study can be found in online repositories. The names of the repository/repositories and accession number(s) can be found below: https://figshare.com/articles/dataset/Data_package/16775728.

## Ethics Statement

The studies involving human participants were reviewed and approved by Faculty of Economics and Business Ethics Committee, University of Groningen. The patients/participants provided their written informed consent to participate in this study.

## Author Contributions

CW primarily drafted and revised the work, while other authors provided substantial edits and feedback to the manuscript. All authors made substantial contributions to the conception, analyses, interpretation of data of this work, and approved the submitted version.

## Conflict of Interest

The authors declare that the research was conducted in the absence of any commercial or financial relationships that could be construed as a potential conflict of interest.

## Publisher’s Note

All claims expressed in this article are solely those of the authors and do not necessarily represent those of their affiliated organizations, or those of the publisher, the editors and the reviewers. Any product that may be evaluated in this article, or claim that may be made by its manufacturer, is not guaranteed or endorsed by the publisher.

## Footnotes

1
https://osf.io/rqup9

2 Between the two sample sources, no significant differences were found in Asian participants’ outcome scores.

3 We initially did not recruit as many participants as expected for Black women. For this reason, to obtain adequate sample size and enough power to run our analyses, we collapsed Latina women with Black women. We reasoned that both groups fall similarly under agency stereotypes. However, doing this downplays the racialized experiences and histories between these groups while dis-acknowledging the complexity of marginalization in the Latina and Hispanic community. Patterns regarding Black women were also found when Latina women were excluded.

4 As pre-registered, the participants also completed measures on the anticipated relevance and success of the intervention, and their feelings of authenticity and leadership intentions. Contrary to our pre-registered hypotheses, the data did not show any differences between the racialized group of women and any of the measures (see “Study 1_Scalar Measures and Null Results” in the Supplementary Materials).

5 The racialization of Latina women is largely varied and can differ immensely from the experiences of Black women. Mohr and Purdie-Vaughns (2015) seminal piece, for example, specifically highlights differences between Black and Latina women; all the while, they call for more scholarship wholly on Latina women. While the groupings of women were changed in the second study, this complexity must be borne in mind when interpreting the results of Study 1.

6 We also measured anticipated intervention success, colorblindness, gender blindness, and belief in meritocracy as potential scalar outcome and exploratory moderator variables. Ultimately, it was not statistically possible to use these variables as moderators in main analyses using Kruskal–Wallis and Dunn tests. Racialized group differences were, however, found and are reported in “Study 2_Exploratory Variables ANOVA and Tukey HSD Analyses” in the Supplementary Materials.

7 Of the nine companies that were randomly selected for manual coding, six organizations focused on *agency* where organizations spotlighted women who demonstrated agency, encouraged cultivating assertiveness, or set targets for female leaders. For example, some testimonials from women of two organizations strongly emphasized the importance of being agentic (e.g., “*I think it is extremely important to take the time to determine what you want and what you need to do to achieve it – and then to be vocal about your ambitions*.”; *“I learned a more effective way of saying no, which helps me cultivate the right relationships and communicate more honestly,” she says, adding that it’s something that has helped her manage her workload better”*).

8 Most of the manually coded organizations mentioned the importance of bringing women together to build networks (*N* = 6); however, this often involved an element of agency. Of the organizations, four linked networking to being assertive by focusing on how agentic women could help other (lower ranked) women become more agentic. To illustrate, an organization mentions on their website that their mentorship program hosts “*female leaders*” who “*help* (…) *to empower girls and women [*…*] to break through long-standing cultural barriers and build rewarding careers of their own*”.

9 In the manual coding we only found two instances where a social group other than gender was mentioned, from which only one brought attention to how these groups intersected.
