# Supplementary material for: Intersectional Invisibility in Women’s Diversity Interventions
Source: Front Psychol. 2022 May 25;13:791572. doi: 10.3389/fpsyg.2022.791572 (PMC9176663; doi:10.3389/fpsyg.2022.791572)
Supplement: Supplementary file 1 [file Data_Sheet_1.zip › Data Sheet 1/Supplemental Material/Study 1_Scalar Measures and Null Results.docx]

**Study 1 Scalar Measures and Results**

**Measures**

For all scaled measures, reverse-scored items were reversed and averaged with other items. Higher values in each measure indicated higher levels of the measured construct.

***Anticipated Authenticity, Relevance, and Success.*** Anticipated authenticity, relevance, and success of the intervention were assessed. Using a 7-point scale (1- strongly disagree, 7- strongly agree), we adapted an authenticity measure(*α=* 0.870) (Jansen et al., 2014, e.g., “I think this leadership program would encourage me to be who I am”, “I would not feel like I could present myself the way I am in this leadership program” (reverse-coded)).

The anticipated program relevance measure (adapted from Wells et al., 1971; *α=* 0.890) included 4 items and participants responded a 7-point scale (1-Strongly Disagree, 7- Strongly Agree; e.g., “This leadership program does not seem like it would be related to my personal circumstances,” “This leadership program does not seem to concern me, or people like me” reverse-coded).

Lastly, anticipated success (*α=* 0.920) was measured using five items and was measured on a 7-point scale (1-Strongly Disagree, 7- Strongly Agree, e.g., I would be happy with how this initiative is implemented, This seems like a worthwhile leadership program).

***Anticipated Leadership Intentions.*** Participants completed two items assessing the extent to which they would pursue higher leadership positions (*α=* 0.950) as a result of the program on a 7-point scale (1- Strongly Disagree, 7- Strongly Agree; Because of this program, I would see myself applying for a higher leadership position in this company, Because of this program, it would be very probable that I would apply for a higher leadership position in this company).

***Open Questions: Reactions and (Anticipated) Leadership Experiences.*** To gain more qualitative insight into the participants’ reactions to the diversity intervention and into participants’ challenges and expectations in the workplace, participants were asked seven open questions about: (1) their reactions to the presented women’s leadership program: “With your previous responses in mind, please list 5 things that you find are important for a leadership program to have for it to be successful for you, personally” and “ In your opinion, based on your experiences as a woman, please provide a detailed description of what YesWomen currently lacks that would be important for you as a participant in this women's leadership program.”, and (2) their previous leadership experiences, or anticipated leadership experiences if they had no prior leadership experience: “Based on your experiences as a woman, how, if at all, would people's stereotypes impact your ability to be effective as a leader?” and “ Based on your experiences as a woman, what unique challenges do you personally expect to face?”.

***Control variables.*** To assess controls in participants’ reactions to the study stimulus, participants completed measures of career ambition ((*α=* 0.890, 10-items; (Gray & O’Brien, 2007), provided information about their workplace (e.g., occupation, work hours), and provided information about their diversity settings such as the diversity composition of their workplace (*α=* 0.910; 3-items; Unzueta & Binning, 2012) and its respective diversity climate(*α=*0.860; 6-items, (Mor Barak et al., 1998)).

**(Null-Effect) Results**

With White women as the reference level, no significant differences were found when regressing racialization as a variable on *anticipated authenticity* (*B*_Asian_= 0.156, *t*_Asian_= 1.283, *p*_Asian_= 0.201; *B*_Black_= 0.240, *t_Black_*= 1.786, *p_Black_*= 0.076), *anticipated personal relevance*(*B*_Asian_= 0.044, *t*_Asian_= 0.482, *p*_Asian_= 0.630; *B*_Black_= 0.060, *t_Black_*= 0.589, *p_Black_*= 0.557), *anticipated intervention success* (*B*_Asian_= 0.099, *t*_Asian_= 0.525, *p*_Asian_= 0.600; *B*_Black_= 0.039 , *t_Black_*= 0.189, *p_Black_*= 0.850), or *anticipated leadership intentions* (*B*_Asian_= 0.129, *t*_Asian_= 0.439 , *p*_Asian_= 0.661; *B*_Black_= 0.037 , *t_Black_*= 0.113, *p_Black_*= 0.910). Racialization was also not a significant predictor of the outcomes when using Asian women as the reference level on the outcomes: *anticipated authenticity* (*B*_Black_= 0.083 , *t_Black_*= 0.553, *p_Black_*= 0.581 )*, anticipated personal relevance (B*_Black_= 0.015 , *t_Black_*= 0.134 , *p_Black_*= 0.894)*, anticipated intervention success*(*B*_Black_= -0.060 , *t_Black_*= -0.260, *p_Black_*= 0.795)*,* and *anticipated leadership intentions* (*B*_Black_= -0.092, *t_Black_*= -0.258, *p_Black_*= 0.797).

Moreover, racialization as a variable did not explain more variance of the outcome variables over that of the control variables used (i.e., diversity climate and composition of participants’’ current workplace, occupation, weekly work hours, and current leadership position): *anticipated authenticity (ΔR^2^=* 0.021*, F*(2, 161)= 1.868, *p*= 0.158) *, anticipated personal relevance(ΔR^2^=*0.003*, F*(2, 161)= 0.220 , *p*= 0.802 )*, anticipated intervention success(ΔR^2^=* 0.002*, F*(2, 161)= 0.138 , *p*= 0.871)*, and anticipated leadership intentions(ΔR^2^=* 0.001*, F*(2, 161)= 0.097 , *p*= 0.908)*.*
