# Supplementary material for: Intersectional Invisibility in Women’s Diversity Interventions
Source: Front Psychol. 2022 May 25;13:791572. doi: 10.3389/fpsyg.2022.791572 (PMC9176663; doi:10.3389/fpsyg.2022.791572)
Supplement: Supplementary file 1 [file Data_Sheet_1.zip › Data Sheet 1/Supplemental Material/Study 1_Survey.docx]

Reactions to Women's Leadership Program

Start of Block: Informed Consent

Q1.1 Thank you for participating in this questionnaire! The aim of this research is to see how participants react to a given women's leadership program.  You will be asked **four** sets of questions about the following: 1. your (anticipated) reactions to a presented women's leadership program, 2. your (anticipated) leadership experiences, 3. your current workplace conditions, 4. and information about yourself.  Your responses will be treated confidentially, and you may withdraw from this study at any point without penalty.

Q1.2 I hereby consent to be a participant in the current research performed by Edwina Wong. 
I agree to take part in the study entitled **Reactions to Women's Leadership Program** and I understand that my participation is entirely voluntary. I understand that my responses will be kept strictly confidential and anonymous. I have the option to withdraw from this study at any time, without penalty, and I also have the right to request that my responses will not be used. The current study will last approximately 20 minutes.  My responses will be treated confidentially and my anonymity will be ensured. Hence, my responses are not identifiable and cannot be linked back to me as an individual. For any questions or comments you may have regarding this research, you may contact c.y.e.wong@student.rug.nl.  

- Yes, I would like to participate (1)
- No, I would not like to participate (2)

Skip To: End of Survey If I hereby consent to be a participant in the current research performed by Edwina Wong.  I agree t... = No, I would not like to participate

End of Block: Informed Consent

Start of Block: Women's Program Advertisement

Q2.1 Imagine that you are an employee of North Edge- a major stationery company- working at a middle management position. At your job, you come across a web pamphlet for a female leadership program. 


The pamphlet (2 pages) can be found on the next page. A timer is set for **one and a half minutes** to read the pamphlet before the "next button" appears, allowing you to continue with the questionnaire. 


Please take this time to read and engage with the pamphlet thoroughly. You will be asked about the information presented, and your evaluations of the pamphlet.

End of Block: Women's Program Advertisement

Start of Block: Women's Program Advertisement 2

Q3.1

Q3.2

Q3.3 Timing

First Click (1)

Last Click (2)

Page Submit (3)

Click Count (4)

End of Block: Women's Program Advertisement 2

Start of Block: Reactions to Women's Program Advertisement

Q4.1 Please imagine that you are working at North Edge, and that you are considering whether you should participate in their YesWomen's leadership program. 


The following questions are about how you think you would feel while considering your participation in YesWomen's leadership program. 


We know you have very limited information to respond to these questions, but that is what we are interested in- your first impressions formed on the basis of limited information.

Q4.2 Click to write the question text

|  | 1. Strongly Disagree (1) | 2. (2) | 3. (3) | 4. (4) | 5. (5) | 6. (6) | 7. Strongly Agree (7) |
| --- | --- | --- | --- | --- | --- | --- | --- |
| If I were to participate in this program, I would feel authentic (1) |  |  |  |  |  |  |  |
| I think this leadership program would encourage me to be who I am (2) |  |  |  |  |  |  |  |
| I think this leadership program would encourage me to express my authentic self (3) |  |  |  |  |  |  |  |
| I would not feel like I could present myself the way I am in this leadership program (4) |  |  |  |  |  |  |  |
| I would feel like I would have to suppress or change myself to fit in with the other participants in this leadership program (5) |  |  |  |  |  |  |  |

Q4.3 Click to write the question text

|  | 1. Strongly Disagree (1) | 2. (2) | 3. (3) | 4. (4) | 5. (5) | 6. (6) | 7. Strongly Agree (7) |
| --- | --- | --- | --- | --- | --- | --- | --- |
| This leadership program does not seem like it would be related to my personal circumstances (1) |  |  |  |  |  |  |  |
| This leadership program seems relevant to me (2) |  |  |  |  |  |  |  |
| This leadership program does not seem to concern me, or people like me (3) |  |  |  |  |  |  |  |
| I think that this program was designed for people like me to be more successful (4) |  |  |  |  |  |  |  |

Q4.4 Please select 2 on the scale.

- 1. Strongly Disagree (1)
- 2. (2)
- 3 (3)
- 4 (4)
- 5 (5)
- 6 (6)
- 7. Strongly Agree (7)

Q4.5 Click to write the question text

|  | 1. Strongly Disagree (1) | 2. (2) | 3. (3) | 4. (4) | 5. (5) | 6. (6) | 7. Strongly Agree (7) |
| --- | --- | --- | --- | --- | --- | --- | --- |
| This leadership program would probably be effective for me (1) |  |  |  |  |  |  |  |
| I would be happy with how this initiative is implemented (2) |  |  |  |  |  |  |  |
| This seems like a worthwhile leadership program (3) |  |  |  |  |  |  |  |
| I would be willing to participate in this leadership program (4) |  |  |  |  |  |  |  |
| If I were interested in getting or succeeding in a leadership position, I would be dissatisfied with this leadership program (5) |  |  |  |  |  |  |  |

Q4.6 Click to write the question text

|  | 1. Strongly Disagree (1) | 2. (2) | 3. (3) | 4. (4) | 5. (5) | 6. (6) | 7. Strongly Agree (7) |
| --- | --- | --- | --- | --- | --- | --- | --- |
| Because of this program, I would see myself applying for a higher leadership position in this company (1) |  |  |  |  |  |  |  |
| Because of this program, it would be very probable that I would apply for a higher leadership position in this company (2) |  |  |  |  |  |  |  |

End of Block: Reactions to Women's Program Advertisement

Start of Block: OQ reactions to Women's Program Advertisement

Q5.1 Please respond to the following open questions. 
 

A timer is set for **two minutes** before the "next button" appears allowing you to continue with the questionnaire. 
 
Please take this time to thoroughly read and respond to the questions.

Q5.2 Timing

First Click (1)

Last Click (2)

Page Submit (3)

Click Count (4)

Q5.3 With your previous responses in mind, please list 5 things that you find are important for a leadership program to have for it to be successful for you, personally. 


For example, what elements would contribute to a safe and pleasant atmosphere for you in a leadership program? What topics should it cover? What kinds of people should be organizing and running the program?

________________________________________________________________

________________________________________________________________

________________________________________________________________

________________________________________________________________

________________________________________________________________

Q5.4 In your opinion, based on your experiences as a woman, please provide a detailed description of what YesWomen currently lacks that would be important for you as a participant in this women's leadership program.

________________________________________________________________

________________________________________________________________

________________________________________________________________

________________________________________________________________

________________________________________________________________

End of Block: OQ reactions to Women's Program Advertisement

Start of Block: (Anticipated) Leadership Experiences

Q6.1 Timing

First Click (1)

Last Click (2)

Page Submit (3)

Click Count (4)

Q6.2 We are now interested in how you would personally feel if you occupied a leadership position (e.g. project manager, department head, supervisor). Therefore, please respond in detail to the following set of open questions. 


IF YOU ARE CURRENTLY A LEADER, OR IF YOU HAVE HAD LEADERSHIP EXPERIENCE, please respond to these questions with this experience in mind. 


A timer is set for **five minutes** before the "next button" appears allowing you to continue with the questionnaire. 


Please take this time to thoroughly read and respond to the questions.

Q6.3 Based on your experiences as a woman, would you anticipate other colleagues or subordinates to be supportive and cooperative with you as a leader?

________________________________________________________________

________________________________________________________________

________________________________________________________________

________________________________________________________________

________________________________________________________________

Q6.4 Based on your experiences as a woman, how, if at all, would people's stereotypes impact your ability to be effective as a leader?

________________________________________________________________

________________________________________________________________

________________________________________________________________

________________________________________________________________

________________________________________________________________

Q6.5 Based on your experiences as a woman, would you anticipate any pushback from anyone because you would be in a leadership position?

________________________________________________________________

________________________________________________________________

________________________________________________________________

________________________________________________________________

________________________________________________________________

Q6.6 Based on your experiences as a woman, what unique challenges do you personally expect to face?

________________________________________________________________

________________________________________________________________

________________________________________________________________

________________________________________________________________

________________________________________________________________

Q6.7 Based on your experiences as a woman, how could leadership programs help you with the issues mentioned in your previous responses?

________________________________________________________________

________________________________________________________________

________________________________________________________________

________________________________________________________________

________________________________________________________________

End of Block: (Anticipated) Leadership Experiences

Start of Block: Work Environment

Q7.1 Now, please respond to some questions about your current work and work environment

| 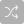 |
| --- |

Q7.2 Please indicate your occupation:

- Management, professional, and related (1)
- Service (2)
- Sales and office (3)
- Education (4)
- Farming, fishing, and forestry (5)
- Construction, extraction, and maintenance (6)
- Production, transportation, and material moving (7)
- Government (8)
- Retired (9)
- Unemployed (10)
- Other, namely (11) ________________________________________________

Display This Question:

If Please indicate your occupation: != Unemployed

| 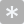 |
| --- |

Q7.3 How many hours a week do you work on average?

________________________________________________________________

Q7.4 Click to write the question text

|  | 1. Strongly Disagree (1) | 2. (2) | 3. (3) | 4. (4) | 5. (5) | 6. (6) | 7. Strongly Agree (7) |
| --- | --- | --- | --- | --- | --- | --- | --- |
| My workplace has a high level of diversity (1) |  |  |  |  |  |  |  |
| I do not consider my workplace to be a diverse organization (2) |  |  |  |  |  |  |  |
| My workplace has a low level of diversity (3) |  |  |  |  |  |  |  |

Q7.5 Click to write the question text

|  | 1. Strongly Disagree (1) | 2. (2) | 3. (3) | 4. (4) | 5. (5) | 6. (6) | 7. Strongly Agree (7) |
| --- | --- | --- | --- | --- | --- | --- | --- |
| At work, I feel I have been treated differently because of my race, sex, religion, or age (1) |  |  |  |  |  |  |  |
| Managers at my work have a track record of hiring and promoting employees objectively, regardless of their race, sex, religion, or age (2) |  |  |  |  |  |  |  |
| Managers at my work give feedback and evaluate employees fairly, regardless of the employees' ethnicity, gender, age, or social background (3) |  |  |  |  |  |  |  |
| Managers at my work make layoff decisions fairly, regardless of factors such as employees' race, sex, age, or social background (4) |  |  |  |  |  |  |  |
| Managers at my work interpret human resource policies (e.g. sick leave) fairly for all employees (5) |  |  |  |  |  |  |  |
| Managers at my work give assignments based on the skills and abilities of employees (6) |  |  |  |  |  |  |  |

End of Block: Work Environment

Start of Block: Personal Information

Q8.1 Lastly, we are interested in some information about you.

Q8.2 Click to write the question text

|  | 1. Strongly Disagree (1) | 2. (2) | 3. (3) | 4. (4) | 5. (5) | 6. (6) | 7. Strongly Agree (7) |
| --- | --- | --- | --- | --- | --- | --- | --- |
| I hope to become a leader in my career field (1) |  |  |  |  |  |  |  |
| When I am established in my career, I would like to manage other employees (2) |  |  |  |  |  |  |  |
| I would be satisfied with just doing my job in a career that I am interested in (3) |  |  |  |  |  |  |  |
| I do not plan to devote energy to getting promoted in the organization or business that I am working in (4) |  |  |  |  |  |  |  |
| When I am established in my career, I would like to train others (5) |  |  |  |  |  |  |  |
| I hope to move up through any organization or business I work in (6) |  |  |  |  |  |  |  |
| Once I finish the basic level of education needed for a particular job, I see no need to continue in school (7) |  |  |  |  |  |  |  |
| I plan on developing as an expert in my career field (8) |  |  |  |  |  |  |  |
| I think I would like to pursue graduate training in my occupational area of interest (9) |  |  |  |  |  |  |  |
| Attaining leadership status in my career is not that important to me (10) |  |  |  |  |  |  |  |

Q8.3 Which of the following best describes your leadership role in your workplace?

- Low-level manager (e.g. supervisor, project manager) (1)
- Middle-level manager (e.g. general managers, branch managers, department managers) (2)
- Top-level manager (e.g. president, vie-president, CEO) (3)
- Not in a managerial or leadership position (4)

Q8.4 Please indicate your gender:

- Male (1)
- Female (2)
- Gender Variant/ Non-Conforming (3)
- Other, namely, (4) ________________________________________________

Display This Question:

If Please indicate your gender: = Male

Or Please indicate your gender: = Female

Q8.5
Cisgender is when your sense of gender identity corresponds with your birth sex. 


Transgender is when your sense of gender identity does not correspond with your birth sex. 

Please indicate if you are cisgender or transgender.

- Cisgender (1)
- Transgender (2)

| 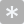 |
| --- |

Q8.6 Please indicate your age:

________________________________________________________________

| 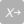 |
| --- |

Q8.7 What is your country of origin?

▼ Afghanistan (1) ... Other (1359)

Skip To: Q8.8 If List of Countries = Other

Q8.8 What is your nationality?

________________________________________________________________

Q8.9 What racial groups apply to you? (options are listed alphabetically- multiple selections are possible)

- Black (1)
- East-Asian (2)
- Hispanic (3)
- Middle-Eastern (4)
- Multi-Racial (5)
- Other Race (6)
- South Asian (7)
- White (8)

Display This Question:

If What racial groups apply to you? (options are listed alphabetically- multiple selections are poss... = Black

Q8.10 You indicated that being **Black** applies to you. 


What ethnic groups apply to you? (multiple selections are possible)

- Caribbean (1)
- African (2)
- Any other Black background. Please specify (3) ________________________________________________

Display This Question:

If What racial groups apply to you? (options are listed alphabetically- multiple selections are poss... = East-Asian

Q8.11 You indicated that being **East-Asian**applies to you. 


What ethnic groups apply to you? (multiple selections are possible)

- Chinese (1)
- Korean (2)
- Japanese (3)
- Any other East-Asian background. Please specify (4) ________________________________________________

Display This Question:

If What racial groups apply to you? (options are listed alphabetically- multiple selections are poss... = Hispanic

Q8.12 You indicated that being **Hispanic** applies to you. 


Please specify which ethnic backgrounds apply to you (e.g. Puerto Rican, Santo Dominican, Cuban):

________________________________________________________________

Display This Question:

If What racial groups apply to you? (options are listed alphabetically- multiple selections are poss... = Middle-Eastern

Q8.13 You indicated that being **Middle-Eastern**applies to you. 


What ethnic groups apply to you? (multiple selections are possible)

- Syrian (1)
- Lebanese (2)
- Palestinian (3)
- Any other Middle-Eastern background. Please specify (4) ________________________________________________

Display This Question:

If What racial groups apply to you? (options are listed alphabetically- multiple selections are poss... = Multi-Racial

Q8.14 You indicated that being **Multi-Racial**applies to you. 


Please specify which ethnic backgrounds apply to you (e.g. British African, American Chinese):

________________________________________________________________

Display This Question:

If What racial groups apply to you? (options are listed alphabetically- multiple selections are poss... = Other Race

Q8.15 You indicated that an **Other Race**applies to you. 


Please specify which race and ethnicity applies to you:

________________________________________________________________

Display This Question:

If What racial groups apply to you? (options are listed alphabetically- multiple selections are poss... = South Asian

Q8.16 You indicated that being **South Asian** applies to you. 


What ethnic groups apply to you? (multiple selections are possible)

- Indian (1)
- Pakistani (2)
- Bangladeshi (3)
- Any other South Asian background. Please specify (4) ________________________________________________

Display This Question:

If What racial groups apply to you? (options are listed alphabetically- multiple selections are poss... = White

Q8.17 You indicated that being **White**applies to you. 


Please specify which ethnic backgrounds apply to you (e.g. Irish, German):

________________________________________________________________

Q8.18 You have reached the end of the survey. If you have feedback to give regarding the questionnaire (e.g. clarity of questions, time), please leave it here.

Thank you again for participating in this questionnaire. Your code is: SEVHW29294

________________________________________________________________

End of Block: Personal Information
