# Supplementary material for: Intersectional Invisibility in Women’s Diversity Interventions
Source: Front Psychol. 2022 May 25;13:791572. doi: 10.3389/fpsyg.2022.791572 (PMC9176663; doi:10.3389/fpsyg.2022.791572)
Supplement: Supplementary file 1 [file Data_Sheet_1.zip › Data Sheet 1/Supplemental Material/Study 1_YesWomen's leadership intervention.docx]

**Study 1 Diversity Intervention for Women Pamphlet**

**
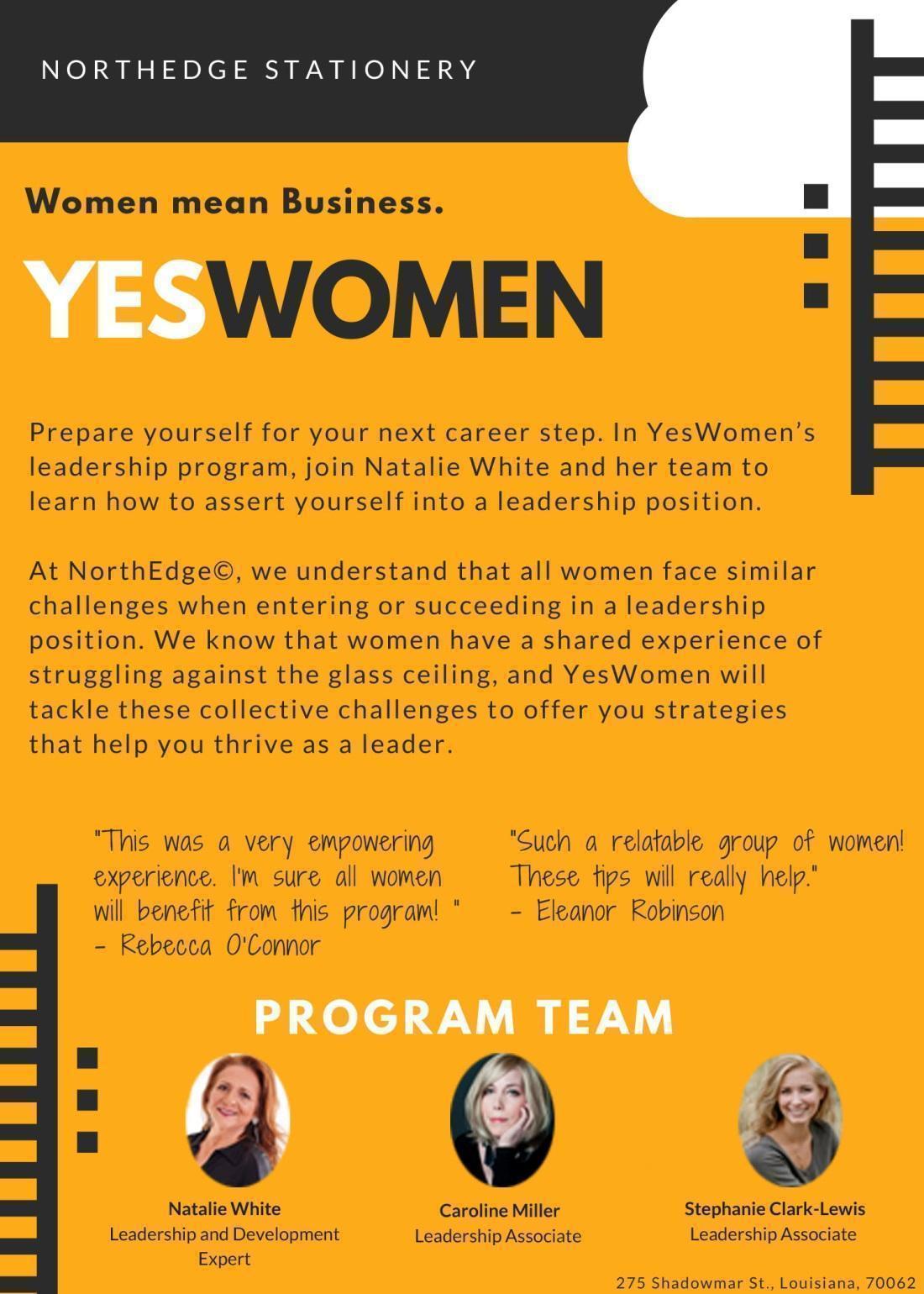
**

*Figure 2.* Single-Target Intervention for Women Pamphlet pt1

*
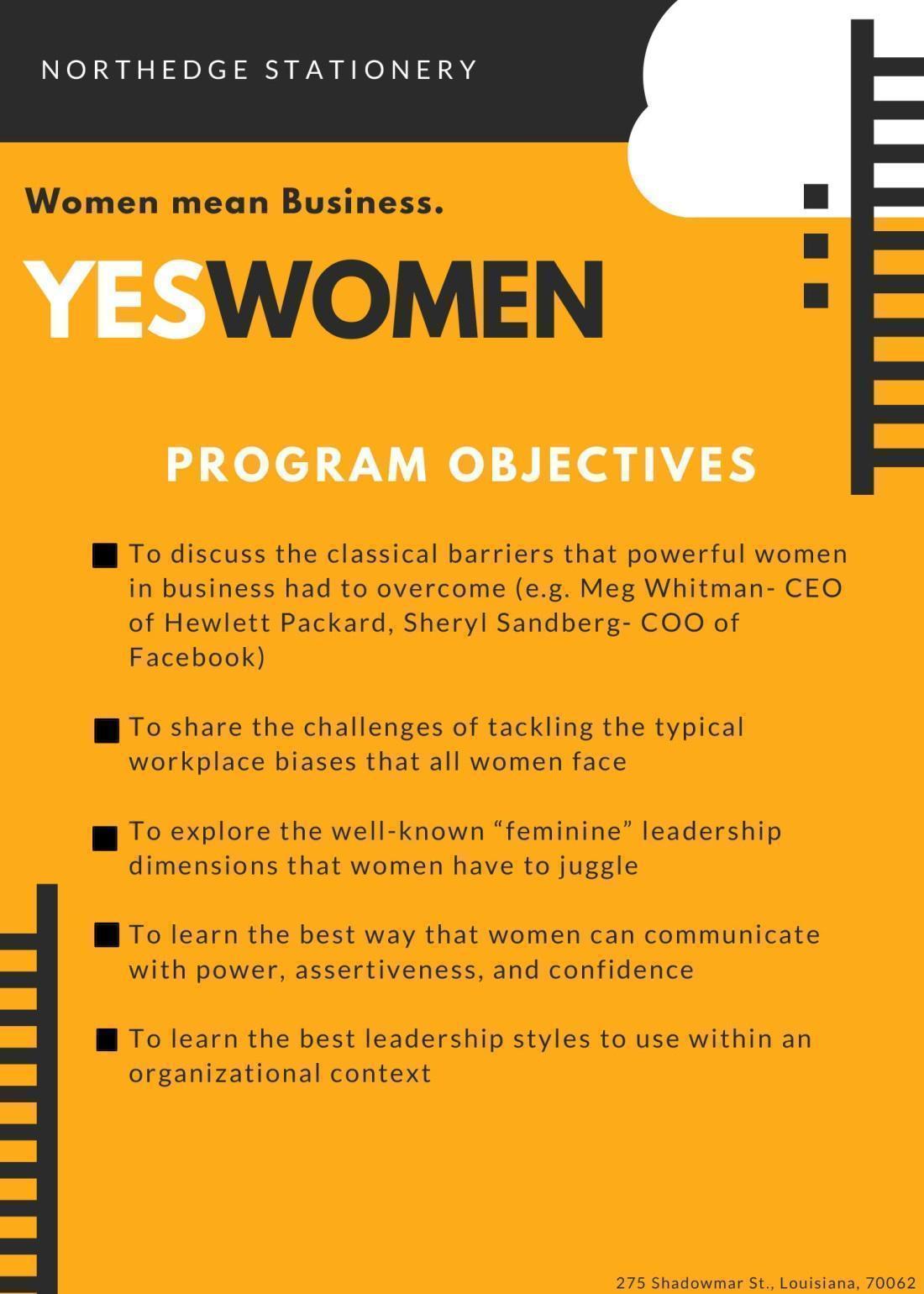
*

*Figure 3.* Single-Target Intervention for women Pamphlet pt2
