# Supplementary material for: Intersectional Invisibility in Women’s Diversity Interventions
Source: Front Psychol. 2022 May 25;13:791572. doi: 10.3389/fpsyg.2022.791572 (PMC9176663; doi:10.3389/fpsyg.2022.791572)
Supplement: Supplementary file 1 [file Data_Sheet_1.zip › Data Sheet 1/Supplemental Material/Study 2_ Aggregated Intervention Needs Results.docx]

**Study 2 Aggregated Intervention Needs Results**

| **Table 10** |  |  |  |  |  | | |  |
| --- | --- | --- | --- | --- | --- | --- | --- | --- |
| *Mean rankings, standard deviations (in brackets), and Dunn tests’ contrasts of each intervention need per racialized group* | | | | | | |  |  |
| Intervention need | Asian women | Black women | White women | Comparison | *z* | *p*. adj | | |
| Intersectional differences | 7.831 (2.197) | 8.405 (2.020) | 6.891 (2.742) | Asian:Black | -2.566 | 0.002 | | |
|  |  |  |  | Asian:White | 1.533 | 0.125 | | |
|  |  |  |  | Black:White | 4.822 | <0.001 | | |
| Networking | 6.079 (2.482) | 5.806 (2.282) | 5.609 (2.322) | Asian:Black | 0.838 | 0.603 | | |
|  |  |  |  | Asian:White | 1.607 | 0.324 | | |
|  |  |  |  | Black:White | 0.611 | 0.541 | | |
| Work-life balance | 5.596 (3.260) | 5.173 (3.130) | 5.805 (3.386) | Asian:Black | 0.848 | 0.594 | | |
|  |  |  |  | Asian:White | -0.444 | 0.657 | | |
|  |  |  |  | Black:White | -1.529 | 0.379 | | |
| Challenges to authority | 6.242 (2.720) | 5.306 (2.624) | 6.220 (2.772) | Asian:Black | 2.451 | 0.021 | | |
|  |  |  |  | Asian:White | 0.134 | 0.894 | | |
|  |  |  |  | Black:White | -2.949 | 0.010 | | |
| Insufficient agency | 7.517 (1.705) | 7.265 (2.129) | 7.889 (2.121) | Asian:Black | 0.533 | 0.594 | | |
|  |  |  |  | Asian:White | -1.920 | 0.082 | | |
|  |  |  |  | Black:White | -2.664 | 0.023 | | |
| Excessive agency | 5.685 (3.356) | 6.255 (3.160) | 6.738 (3.270) | Asian:Black | -1.153 | 0.249 | | |
|  |  |  |  | Asian:White | -2.629 | 0.026 | | |
|  |  |  |  | Black:White | -1.275 | 0.304 | | |
