# Supplementary material for: Intersectional Invisibility in Women’s Diversity Interventions
Source: Front Psychol. 2022 May 25;13:791572. doi: 10.3389/fpsyg.2022.791572 (PMC9176663; doi:10.3389/fpsyg.2022.791572)
Supplement: Supplementary file 1 [file Data_Sheet_1.zip › Data Sheet 1/Supplemental Material/Study 2_Exploratory Variables ANOVA and Tukey HSD Analyses.docx]

**ANOVA and post-hoc Tukey HSD analyses for exploratory variables**

Across the racialized groups, the ANOVA tests (see Table 8) indicated significant differences in the anticipated intervention success in an intervention that incorporated participants’ top 3 prioritized rankings, participants’ belief in colorblindness, and participants’ belief in meritocracy. When examining the breakdown of these results (see Table 9), Black women most significantly anticipated more intervention success should their prioritized rankings were incorporated in the diversity intervention for women compared to White women and Asian women. Moreover, Black women significantly reported lower scores in belief in colorblindness. Interpreting this result along with the main analyses, this pattern of results suggest that Black women indicate more centrality to racialization than either White women or Asian women in this sample. Lastly, White women showed the highest scores on belief in meritocracy, followed by Asian women and Black women respectively.

| **Table 8** |  |  |  |  |
| --- | --- | --- | --- | --- |
| *ANOVA analyses results of the exploratory variables by racialized group* | | | | |
|  | Intervention success | Colorblindness | Belief in meritocracy | Gender blindness |
| *F* | 9.942 | 19.39 | 27.53 | 1.846 |
| *df* | (2, 486) | (2, 486) | (2, 486) | (2, 486) |
| *p* | > 0.001 | > 0.001 | > 0.001 | 0.159 |

| **Table 9** |  |  |  |  |  | | |  |
| --- | --- | --- | --- | --- | --- | --- | --- | --- |
| *Mean rankings and Tukey HSD contrasts of each intervention need per racialized group* | | | | | | |  |  |
| Intervention need | Asian women *(x̅)* | Black women *(x̅)* | White women (*x̅*) | Comparison | *CI* | *p*. adj | | |
| Intervention success | 5.421 | 5.811 | 5.305 | Asian:Black | [-0.723, -0.054] | 0.018 | | |
|  |  |  |  | Asian:White | [-0.161, 0.392] | 0.587 | | |
|  |  |  |  | Black:White | [0.239, 0.772] | >0.001 | | |
| Colorblindness | 3.023 | 2.561 | 3.228 | Asian:Black | [0.144, 0.781] | 0.002 | | |
|  |  |  |  | Asian:White | [0.467, 0.057] | 0.157 | | |
|  |  |  |  | Black:White | [-0.920, -0.415] | >0.001 | | |
| Belief in meritocracy | 3.160 | 2.689 | 3.665 | Asian:Black | [0.680, 0.875] | 0.017 | | |
|  |  |  |  | Asian:White | [-0.837, -0.172] | 0.001 | | |
|  |  |  |  | Black:White | [-1.296, -0.656] | >0.001 | | |
| Gender blindness | 4.748 | 4.673 | 4.835 | Asian:Black | [-0.184, 0.334] | 0.776 | | |
|  |  |  |  | Asian:White | [-0.300, 0.127] | 0.606 | | |
|  |  |  |  | Black:White | [-0.367, 0.044] | 0.156 | | |
